# Case report: Severe combined immunodeficiency with ligase 1 deficiency and Omenn-like manifestation

**DOI:** 10.3389/fimmu.2022.1033338

**Published:** 2022-10-19

**Authors:** Nel Dabrowska-Leonik, Agata Karolina Pastorczak, Katarzyna Bąbol-Pokora, Katarzyna Bernat-Sitarz, Barbara Piątosa, Edyta Heropolitańska-Pliszka, Magdalena M. Kacprzak, Krzysztof Kalwak, Katarzyna Gul, Mirjam van der Burg, Marek Ussowicz, Malgorzata Pac

**Affiliations:** ^1^ Department of Immunology, Children’s Memorial Health Institute, Warsaw, Poland; ^2^ Department of Pediatrics, Oncology and Hematology, Medical University of Lodz, Lodz, Poland; ^3^ Histocompatibility Laboratory, Children’s Memorial Health Institute (IPCZD), Warsaw, Poland; ^4^ MedGen Medical Center, Warsaw, Poland; ^5^ Department of Paediatric Bone Marrow Transplantation, Oncology and Hematology, Wroclaw Medical University, Wroclaw, Poland; ^6^ Department of Pediatrics, Leiden University Medical Center (LUMC), Leiden, Netherlands

**Keywords:** ligase 1, immunodeficiency, Omenn-like, hematopoietic stem cell transplantation (HCST), Immunoglobilins

## Abstract

DNA ligase I deficiency is an extremely rare primary immunodeficiency with only 6 patients reported in the literature. Most common manifestations include radiosensitivity, macrocytic anemia, lymphopenia with an increased percentage of gamma-delta T cells, and hypogammaglobulinemia requiring replacement therapy. Two-month-old girl with delayed development, T-B-NK+ SCID, and macrocytic anemia presented features of Omenn syndrome. Whole exome sequencing revealed two novel, heterozygous variants (c.2312 G>A, p.Arg771Gly and c.776+5G>T, p.Pro260*) in the LIG1 gene (NM_000234.1). Hematopoietic stem cell transplantation from a fully matched unrelated donor was performed at the age of 4 months using GEFA03 protocol. Mixed donor-recipient chimerism was observed, with 60-70% chimerism in the mononucleated cell compartment and over 90% in T-lymphocyte compartment, but autologous myeloid recovery. Stable CD4+ and CD8+ T-cell counts above 200/µL were achieved after 2 months, but the patient remained transfusion-dependent. Despite satisfactory immunological reconstitution, the second transplantation due to constitutional hemolytic defect has been considered. In light of possible re-transplantation, an issue of optimal conditioning protocol with sufficient myeloid engraftment is important. For the first time Omenn syndrome is described in a compound heterozygote carrying two the novel variants p.Arg771Gly and p.Pro260* in the LIG1 gene. Patients diagnosed with SCID and Omenn syndrome showing macrocytic anemia, should be screened for DNA ligase I deficiency.

## Introduction

DNA ligase I deficiency is an extremely rare autosomal recessive primary immunodeficiency, caused by mutations in *LIG1* gene located on chromosome 19. As result, Okazaki fragments are improperly catalyzed during cell replication and single-strand DNA damage repair. The disease is associated with a diverse spectrum of clinical symptoms beginning in infancy or early childhood (1). An increased susceptibility to infections, macrocytic anemia, lymphopenia, increased percentage of γδ T cells, hypogammaglobulinemia requiring replacement therapy, and increased sensitivity to DNA damaging agents have been reported in all patients (2–4). Most of the only six reported cases demonstrated normal mental development. Growth retardation or failure to thrive and delayed or absent sexual maturation have been observed in two patients. Phenotype of one of the patients with delayed sexual maturation resembled Bloom syndrome and the patient died at the age of 19 years due to lymphoma (2). Fibroblasts from this patient showed an increased sensitivity to DNA damage caused by alkylating agents and ionizing and UV radiation (5, 6). Five remaining patients had normal physical and mental development (4). Severe combined immunodeficiency (SCID) was diagnosed in two cases and treated with allogeneic hematopoietic stem cell transplantation (HSCT) with reduced intensity conditioning regimen (3). Omenn syndrome was suspected in one patient, but skin biopsy did not confirm the diagnosis (3, 4).

## Case description

**Figure 1:**
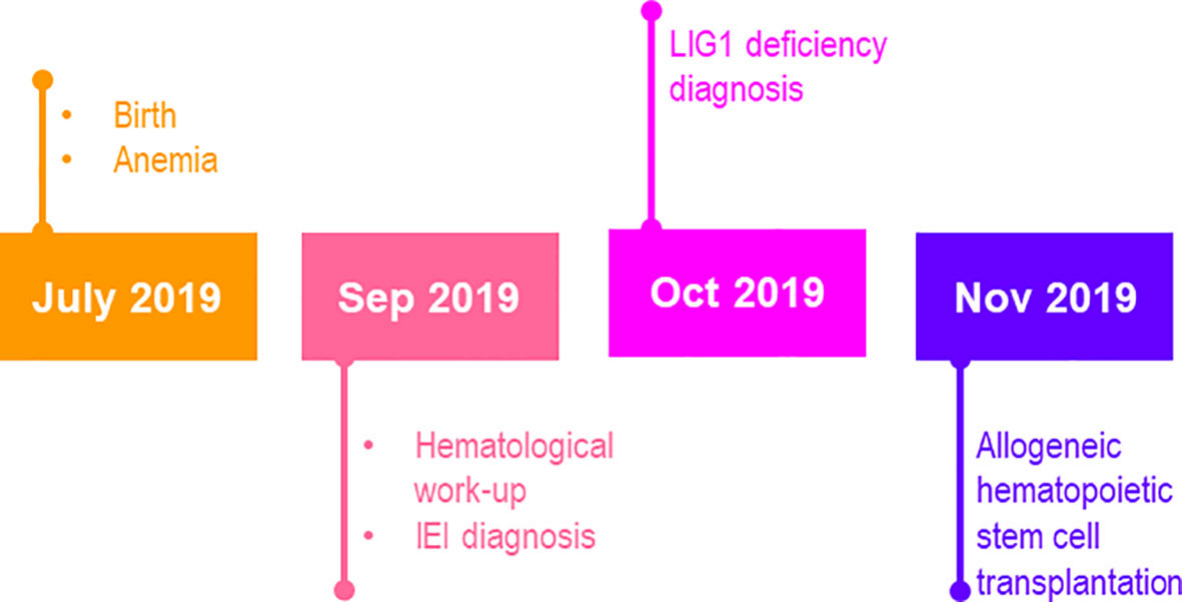
The timeline of case report.

**Figure 2:**
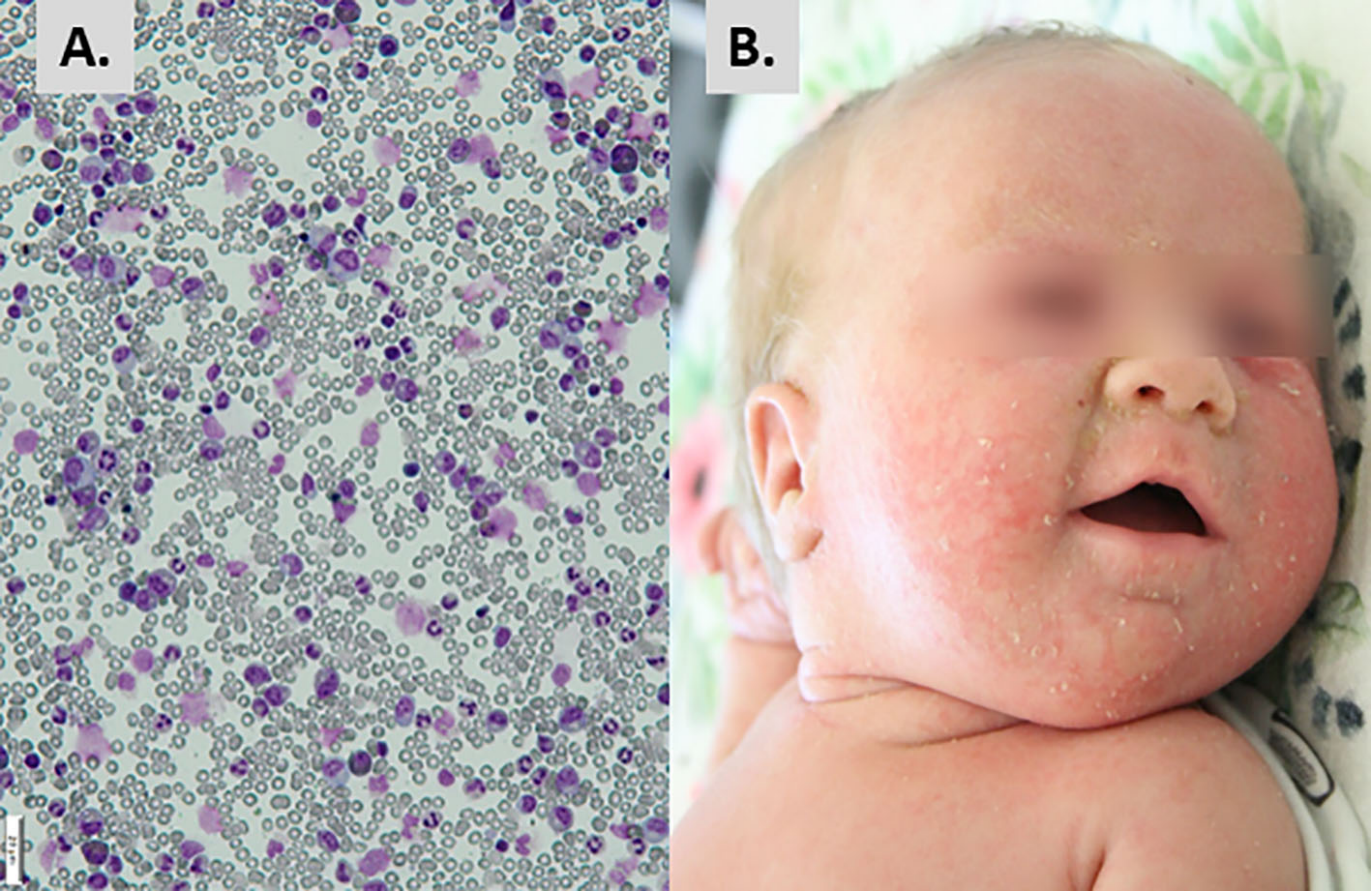
Initial presentation and diagnostic hallmarks. **(A)** Hypercellular bone marrow with suppressed erythroblastic lineage. **(B)** Erythematous-exfoliative skin lesions.

A female infant was born at 35 weeks of gestation due to the first uncomplicated pregnancy of 28 y.o. mother by vaginal delivery with birth weight 1950 g and the 10 minutes Apgar score 6. Parents were healthy, non-consanguineous. After birth she demonstrated respiratory problems and hepatosplenomegaly. Blood tests revealed macrocytic anemia since the first day of life, with hemoglobin concentration 6.9 G/dL at birth date [normal range for the age, (N: 14.9-23.7 G/dL], mean corpuscular volume (MCV) 133 fL [N: 100-125 fL], thrombocytopenia in the first week of life (minimum platelet count 49x10^3^/µL), and lymphopenia since the third day of life (4-16%; 0.2 – 1.3x10^3^/µL). Due to respiratory failure probably of non-infectious cause, she was treated with a non-invasive positive pressure ventilation for one day, with nasal continuous positive airway pressure for the next 3 days. Infection markers and microbiological diagnostics were negative but newbborn was treated with empirical antibiotics (ampicillin and amikacin). Due to anemia, the child received multiple transfusions of irradiated, filtered, packed red blood cells and 10 doses of erythropoietin. Platelet concentrate was transfused once due to thrombocytopenia. Intravenous immunoglobulins were administered twice without serum immunoglobulins level tests (see the timeline in **Figure 1**). The girl was discharged from the neonatal unit at 32 days of age, but after a week, at the age of 5 weeks, she was re-admitted due to anemia (Hb 7.3 G/L) and lymphopenia 0.7x10^3^/µL) with erythematous papular rash not observed in neonatal period. Vitamin B12 and folate deficiency have been excluded. Flow cytometry revealed deep lymphopenia with T lymphocyte count 30 cells/µL, CD19+ B lymphocytes 93 cells/µL, and NK CD3-CD56.CD16+ 196 cells/µL. Hypercellular bone marrow with sparse erythroblastic and a reduced lymphocytic line, without signs of malignant proliferation, were found in bone marrow biopsy (**Figure 2A**). Based on the obtained laboratory results severe combined immunodeficiency was suspected.

The infant was admitted to the Immunology Department, Children’s Memorial Health Institute, Warsaw, at the age of 8 weeks, in moderate general condition. The patient showed generalized erythematous-small lobular skin eruptions, with exfoliation located mainly on the face (**Figure 2B**), conjunctivitis of *Staphylococcus aureus* origin, and enlarged cervical and inguinal lymph nodes of up to 1 cm diameter. The size of spleen and liver were within the age-related normal ranges. The body length was below 3 percentile in relation to percentile growth charts for preterm infants at gestational age 32-37 weeks, but the body weight and head circumference were proportional to length. Systemic antibiotic therapy, palivizumab, and intravenous immunoglobulins (IVIG) have been introduced to treatment, while anti-viral, anti-fungal, anti-*Pneumocytis jiroveci* pneumonia (PJP) prophylaxis was continued. Due to anemia, the girl required red blood cell transfusions approximately every 2 weeks. During hospitalization, mild but chronic diarrhea and erosions around the anus were observed with a transient rotavirus positive stool result. Immunological tests were repeated and based on results severe combined immunodeficiency with decreased number of T, B and NK cells was diagnosed (**Table 1**).

**Table 1:** Laboratory results at the time of diagnosis.

Parameter	Result	Normal range for the age
CD45+	313 cells/μl	(3800-8100 cells/μl)
CD3+	106 cells/μl	(2200-5500 cells/μl)
CD3+CD4+	87 cells/μl	1400-4200 cells/μl
CD3+CD8+	5 cells/μl	400-1400 cells/μl
CD19+	107 cells/μl	700-1800 cells/μl
CD56+	84 cells/μl	200-980 cells/μl
CD31+CD45RA+/CD3+CD4+	0%	50-74%
γδT%	18,9%	3,2-4,9%
IgG	5.96 g/l (1 day after IVIG)	3.36-10.5 g/l
IgM	<0.04 g/l	0.21-0.51 g/l
IgA	<0.07 g/l	<0.06-0.07 g/l
IgE	<2.00 kIU/L	
Mitogen response tests		
PHA	1705 ± 56 SI 11.0	>16000 cpm >65
anty-CD3	2494 ± 159 cpm, SI 16	>15000 cpm >60
Pansorbin	20646 ± 224 cpm SI 133	>2000 cpm >5
Microbiology		
Nasal swab	*Staphylococcus aureus MSSA, Klebsiella pneumoniae*	
Throat swab	Physiological flora	
Rectal swab	Physiological flora	
Fecal culture	Physiological flora, *Salmonella*, *Shigella* negative, Norovirus, adenovirus negative, rotavirus positive for 4 days,	
Conjunctival culture	*Staphylococcus aureus MSSA*	
Swab of the external auditory canal	*Staphylococcus aureus MSSA, Escherichia coli* ESBL/-/	
PCR in whole blood for CMV, EBV, HSV-1, HSV-2, Enterovirus, Human paraechovirus, HHV-6, HHV-7, Parvovirus B19	negative	
Serum Galactomannan, mannan	negative	

CMV, Cytomegalovirus; EBV, Epstein-Barr virus; HHV-6, Human herpesvirus 6; HHV-7, Human herpesvirus 7; HSV-1, Herpes simplex virus type 1; HSV-2 – Herpes simplex virus type 2; IgA – immunoglobulin A; IgE, immunoglobulin E; IgG, immunoglobulin G; IgM, immunoglobulin M; MSSA, methicillin-susceptible Staphylococcus aureus; PCR – polymerase chain reaction; PHA, phytohemagglutinin.

**Figure 3:**
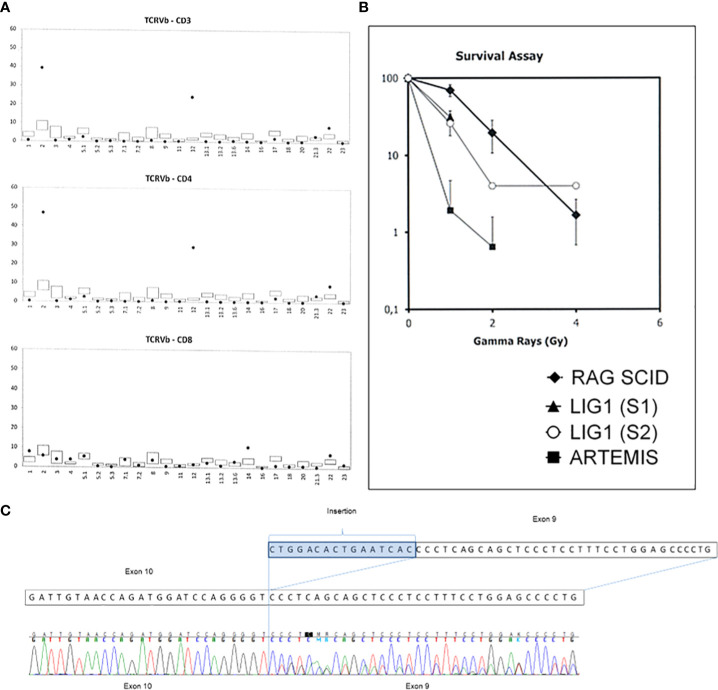
Diagnostic key-tests in the LIG1 deficiency patient. **(A)** Distribution of Vβ chain of the TCR receptor is oligoclonal with a significantly increased percentage of Vβ2 and Vβ12 chains among CD4 lymphocytes. **(B)** Clonogenic survival assay results. Fibroblasts cultured from skin biopsies of the patient and an Artemis-deficient SCID patient were exposed to increasing dose of irradiation and the percentage survival (axe Y) was determined after 8 days. S1- sample 1, S2- sample 2. RAG1 SCID patient sample was used as a control. **(C)** The cDNA sequencing results showing the splice site variant in the LIG1 which leads to an insertion of sixteen nucleotides in the exon 9 resulting in a shift of a reading frame and a premature protein termination at position 260.

Omenn syndrome was suspected due to erythematous-exfoliative skin lesions, peripheral lymphadenopathy, eosinophilia (7020/μl), despite low concentration of IgE (<2.00 kIU/L), and oligoclonal distribution of TCR Vβ chain (**Figure 3A**). Feto-maternal chimerism was excluded. Fibroblasts cultured from skin biopsy showed an intermediate sensitivity to ionizing radiation (**Figure 3B**) in comparison to fibroblasts from a patient with radiosensitive SCID due to Artemis deficiency. Histopathological evaluation of skin biopsy was not carried out due to small surface of typical skin lesions present mainly on head and face. Combined prednisone and cyclosporine A immunosuppressive treatment resulted in gradual improvement of skin condition and reduction of eosinophilia.

Whole exome sequencing of the patient’s DNA was performed. The libraries were prepared using Twist Human Core Exome Plus Kit (Twist Bioscience) and sequenced on Illumina platform with a 100x mean mapped read depth. The results of genomic testing revealed presence of two rare heterozygous variants in the *LIG1* gene (NM_000234.1): a missense variant of uncertain significance c.2312 G>A, p.Arg771Gly with high pathogenicity scores, including DANN score (7) reaching 0.9995, and a likely pathogenic splice variant c.776+5G>T, p.Pro260*. In order to verify whether the c.776 +5G>T variant affected mRNA splicing, direct cDNA sequencing was executed. It was found that the splice-variant resulted in an insertion of 16 nucleotides in intron 9, leading to a premature protein synthesis termination at position 260 (**Figure 3C**). Direct sequencing of DNA of the child and her parents in search of the presence of both variants of *LIG1* was subsequently performed (**Supplementary Table 1**). It was found that each of the parents was a carrier of a single *LIG1* mutation, which confirmed that both *LIG1* variants are located in the proband on separate alleles. Datasets are submitted to the European Variation Archive (EVA) repository (https://www.ebi.ac.uk/eva/?eva-study=PRJEB*****) and are publicly accessible under project number PRJEB56316, accession numbers ERZ14199911 at EMBL-EBI.

Due to the immunological features of severe combined immunodeficiency, the girl was referred for HSCT from an unrelated donor. At the age of 4 months, the girl was transplanted from a fully 10/10 HLA allele-matched unrelated donor using GEFA03 protocol, with intravenous busulfan at 0.5 mg/kg body weight (BW) given twice daily on days -4 and -3 (total dose 2 mg/kg), fludarabine i.v. at 30 mg/m2 daily from day -9 to day -4 (total dose 180 mg/m2), cyclophosphamide i.v. at 20 mg/kg daily on days -3 and -2 (total dose 40 mg/kg), and i.v. antithymocyte globulin (ATG)-Fresenius at 20 mg/kg BW daily from day -3 to day -1 (8).

Prophylaxis of a graft vs host disease (GVHD) was composed of cyclosporin A from -1 day and methotrexate on days +1, +3, +6. Peripheral blood progenitor cells in 14.67 x 10^6^ CD34+ cells/kg BW dose were transplanted. Granulocytes recovered on +8 day after HSCT. From day +28, an isolated acute stage 2 skin GVHD in form of fine-grained rash on skin of the trunk and abdomen was diagnosed and treated with methylprednisolone. The patient developed mixed hematopoietic chimerism that stabilized at the level of 60-70% mononuclear cells of donor origin and more than 90% lymphocytes of donor origin (**Supplemental Figure 1A**). Immune reconstitution was regularly monitored (lymphocyte subset analysis results are shown on **Supplemental Figure 1B**), and the patient achieved stable CD4+ and CD8+ T-cell counts above 200 cells/mL after 2 months (**Supplemental Figure 1C**).

At the time of preparing of the manuscript, the girl has been followed-up for two years after HSCT. She is slightly delayed in physical and motor development. B-cell lymphopenia is observed permanently and the patient needs immunoglobulin therapy, as well as occasionally - red blood cells transfusions. The direct and indirect antiglobulin tests were negative, LDH activity, bilirubin direct and indirect results were within normal range. Due to anemia, the second transplantation has been considered, but the patient’s parents did not consent to re-transplantation.

## Discussion

The reported patient showed unique clinical features caused by presence of combined heterozygous mutation within the *LIG1* gene. The splice variant c.776+5G>T, p.Pro260* resulted in the insertion of 16 nucleotides in the intron 9 of *LIG1* leading to premature protein termination at position 260, and in consequence - to absence of functional domains of the protein. The missense variant (c. 2311G> T, p.Arg771Gly, R771G), which affected the structure of catalytic oligonucleotide/oligosaccharide binding-fold domain (OBD), was caused by defect located at the same position as reported in 4 other patients with *LIG1* deficiency (R771W), but associated with a different amino acid change (9, 10). However, both R771W and R641L variants decrease the activity of the DNA ligase (11, 12).

The biological function of ligase 1 in the replication and DNA repair is associated with sealing of Okazaki fragments during replication and catalyzing the ultimate ligation step of DNA repair (12). The cellular functions of LIG1 are mediated through its non-catalytic N-terminal domain (amino acids 1-261), that contains the nuclear localization signal and participates in protein–protein interactions (13).

DNA ligase I deficiency causes an extremely rare IEI with a diverse spectrum of clinical symptoms affecting not only immunity, but also growth, psychomotor development, and production of blood cells. The course of the disease in the reported patient included severe anemia requiring multiple red blood cell transfusions since the first hours of life. It is unclear why mutations in the *LIG1* gene cause macrocytic anemia and other manifestations extending beyond immune deficiency, and whether any genotype-phenotype correlation exists. Macrocytic anemia probably results from an impaired DNA synthesis in hematopoietic precursor cells, yet in contrast to Fanconi anemia - it does not lead to bone marrow failure, but to hemolysis moderately compensated by reticulocyte production. Impaired DNA synthesis may also affect other rapidly dividing hematopoietic cells.

An IEI was for the first time suspected at the age of 8 weeks when absent T- and low B-lymphocyte counts were noted during work-up for constitutional hemolytic anemia. The diagnosis of T-B-NK+ SCID was similar to two other reported boys (3).

The presence of erythematous-exfoliating skin lesions suggestive of Omenn syndrome, due to uncontrolled proliferation of autologous T lymphocytes was a unique feature in the reported patient. The diagnosis was supported by clinical criteria of the ESID Registry, which state that probable diagnosis of Omenn’s syndrome may be made when exfoliative erythroderma occurs in the first year of life and it is accompanied by at least one clinical symptom: lymphoproliferation, failure to thrive, chronic diarrhea, recurrent pneumonia, eosinophilia or elevated IgE with T-cell deficiency (low naive cells, reduced proliferation, oligoclonality), and with maternal engraftment and HIV infection excluded (14). Although there was no histopathological proof for such diagnosis, our patient was successfully treated as an Omenn syndrome with prednisone and cyclosporine: skin lesions, lymphadenopathy, and diarrhea disappeared, and the number of eosinophils normalized.

The essential consequence of *LIG1* defect is an impaired DNA repair mechanism. The reported patient’s cells demonstrated an increased sensitivity to ionizing, UV radiation and DNA damaging agents *in vitro*, as in the first reported patient, or only to methyl methanosulphate, as in two SCID patients (4). Fibroblasts of our patient were sensitive to ionizing radiation and although sensitivity to other DNA damaging agents was not verified, it was considered as decisive information when HSCT procedure was planned. Based on the guidelines for HSCT in patients with radiosensitivity, and center experience - a reduced intensity conditioning protocol was administered. The peri-transplant period was relatively uneventful, mucosal toxicities were mild, and the patient was fed orally. However, the modified German Fanconi anemia protocol did not show sufficient myeloablative potential, as observed in ataxia-telangiectasia and Nijmegen breakage syndrome (15, 16).

Decreased pretransplant NK-cell counts can point out to exhaustion of common lymphoid progenitor compartment. At time of transplantation, the patient was profoundly lymphopenic and NK cell counts were not likely to affect the engraftment. It can not be ruled out, that suboptimal myelosuppression resulted in autologous recovery of NK cells and accelerated elimination of donor derived hematopoiesis. B- and NK-cell chimerism was not evaluated. The myeloid chimerism evaluation in CD15 positive cells was performed only once. All other chimerism measurements were carried up in mononuclear cells or in T-lymphocytes. It can be hypothesized, that vestigial donor hematopoiesis and myelopoesis (ca. 2% of all CD15 cells) are responsible for production of NK cells, and lack of competition from autologous lymphoid.

The reported experience with HSCT in *LIG1* deficiency suggested effectiveness of minimal intensity conditioning, but this was not confirmed in case of our patient. Both previously reported boys who underwent HSCT in Great Ormond Street Hospital (GOSH) from family donors,received minimal intensity conditioning: fludarabine 150 mg/m2, cyclophosphamide 1000 mg/m2, alemtuzumab 1 mg/kg and YTH 24/54 (anti-CD45 monoclonal antibodies) 800 μg/kg. Although the first patient received additionally YTH 24/54 anti-CD45 monoclonal antibodies in reduced dose due to adverse reaction during infusion, he achieved multilineage full donor chimerism. The second patient demonstrated low level B-cell engraftment, was transfusion-dependent, and remained on immunoglobulin replacement therapy. He also needed blood transfusions and chelation therapy (3).

Optimal conditioning protocol with sufficient myeloid engraftment is important in case of considered re-transplantation, but there is not enough data to determine the tolerance to chemotherapy regimens. In addition, despite limited long-term follow-up of patients with *LIG1* deficiency, an elevated risk of cancer incidence should be considered.

## Conclusions

DNA ligase I deficiency should be considered in patients diagnosed with SCID associated with macrocytic anemia and features of Omenn syndrome. For the first time Omenn syndrome is described in a compound heterozygote carrying two novel variants p.Arg771Gly and p.Pro260* in the *LIG1* gene. HSCT is an option to cure LIG1 deficiency, however myeloid chimerism is required to control anemia and for sustained B-cell function, and an intensity of conditioning regimen remains questionable.

## Data availability statement

The data presented in the study are deposited in the European Variation Archive (EVA) repository (https://www.ebi.ac.uk/eva/?eva-study=PRJEB56316), project number PRJEB56316, accession numbers ERZ14199911.

## Ethics statement

Ethical review and approval was not required for the study on human participants in accordance with the local legislation and institutional requirements. Written informed consent to participate in this study was provided by the participants’ legal guardian/next of kin. Written informed consent was obtained from the individual(s), and minor(s)’ legal guardian/next of kin, for the publication of any potentially identifiable images or data included in this article.

## Author contributions

ND-L, MP designed the concept of the manuscript. ND-L, AP, KB-P, BP, MU, MP wrote the manuscript with contribution from all co-authors. BP did the immunological studies AP, KB-P, MK did the genetic analysis. MB did the radiosensitivity of fibroblasts. ND-L, KB-S, EH-P, KG, KK, MU, MP contributed to clinical data collection and critical review. All authors contributed to the article and approved the submitted version.

## Conflict of interest

The authors declare that the research was conducted in the absence of any commercial or financial relationships that could be construed as a potential conflict of interest.

## Publisher’s note

All claims expressed in this article are solely those of the authors and do not necessarily represent those of their affiliated organizations, or those of the publisher, the editors and the reviewers. Any product that may be evaluated in this article, or claim that may be made by its manufacturer, is not guaranteed or endorsed by the publisher.
