# Supplementary material for: Case report: Severe combined immunodeficiency with ligase 1 deficiency and Omenn-like manifestation
Source: Front Immunol. 2022 Oct 19;13:1033338. doi: 10.3389/fimmu.2022.1033338 (PMC9626757; doi:10.3389/fimmu.2022.1033338)
Supplement: Supplementary file 1 [file DataSheet_1.docx]

Supplementary Table 1. Sequences of primers used for direct LIG1 gene sequencing

| **Region** | **Forward primer** | **Reverse primer** |
| --- | --- | --- |
| E9 | 5’ ACCGTCTGAGCTCTCTCACC | 5’ CTACGGGGATCAGACTGAGG |
| E24 | 5’ AGTTCCCTTCAGACTTACTTCTAG | 5’ ATGTGAGCCACCACTATTTGTA |





Supplementary Figure 1. Posttransplant results: donor chimerism (A), immune reconstitution in lymphocyte subsets (B), and lymphocyte count (C).
